# Entropy and Gaussian Filter-Based Adaptive Active Contour for Segmentation of Skin Lesions

**DOI:** 10.1155/2022/4348235

**Published:** 2022-07-19

**Authors:** Saleem Mustafa, Muhammad Waseem Iqbal, Toqir A. Rana, Arfan Jaffar, Muhammad Shiraz, Muhammad Arif, Samia Allaoua Chelloug

**Affiliations:** ^1^Department of Computer Science, Superior University, Lahore 54600, Pakistan; ^2^Department of Software Engineering, Superior University, Lahore 54600, Pakistan; ^3^Department of Computer Science and IT, The University of Lahore, Lahore 54000, Pakistan; ^4^School of Computer Sciences, Universiti Sains Malaysia, Penang 11800, Malaysia; ^5^Department of Computer Science, Federal Urdu University of Arts, Science & Technology, Islamabad 44000, Pakistan; ^6^Department of Information Technology, College of Computer and Information Sciences, Princess Nourah bint Abdulrahman University, P. O. Box 84428, Riyadh 11671, Saudi Arabia

## Abstract

Malignant melanoma is considered one of the deadliest skin diseases if ignored without treatment. The mortality rate caused by melanoma is more than two times that of other skin malignancy diseases. These facts encourage computer scientists to find automated methods to discover skin cancers. Nowadays, the analysis of skin images is widely used by assistant physicians to discover the first stage of the disease automatically. One of the challenges the computer science researchers faced when developing such a system is the un-clarity of the existing images, such as noise like shadows, low contrast, hairs, and specular reflections, which complicates detecting the skin lesions in that images. This paper proposes the solution to the problem mentioned earlier using the active contour method. Still, seed selection in the dynamic contour method has the main drawback of where it should start the segmentation process. This paper uses Gaussian filter-based maximum entropy and morphological processing methods to find automatic seed points for active contour. By incorporating this, it can segment the lesion from dermoscopic images automatically. Our proposed methodology tested quantitative and qualitative measures on standard dataset dermis and used to test the proposed method's reliability which shows encouraging results.

## 1. Introduction

Melanoma is one form of skin cancer. Recent researches show that melanoma is the most dangerous kind of skin cancer. An important reason is that melanoma affects about 75% of death reported skin cancer. In 2017, research shows that around 9,480 out of 76,690 melanoma patients died by the cause of melanoma in the USA [1]. In addition, 1 in 74 males and 1 in 90 females may infect with melanoma in their life in Canada. Previous research also shows that for non-Hispanic American white people, occurrence rates have increased yearly by around 3%. For grown people ages 15 and 30, melanoma is considered the most popular identifiable type of cancer [2]. Discovering melanoma in the early stage will increase the probability from 5 years to 96% of remaining alive. Still, in case of finding it in the very advanced stage, that percentage will decrease to 5% [2]. The recovery percentage is affected, and the melanoma treatment cost in the advanced stage is 30 times more than the cost of melanoma treatment in the early stages. Dermoscopic device helps physicians view the lesion features more clearly than the naked eye [3].

Diagnosis of skin lesion and classification has been an activity in most areas of medical science research for quite a while now. Melanoma is one of the lethal and rare forms of skin lesion. Due to this type of lesion, the death rate is three times more than all other skin lesions. It is common, especially among white skin people. Diagnosis of malignant melanoma in the initial stage increases the survival rates and is curable. Unluckily, screening all the patients with a skin lesion for dermatologists is very difficult, and the cost of treating this type of skin lesion is very high. Working out the dermoscopic clinical features like lesion borders, pigment networks, and the color of melanoma is a vital step for dermatologists, who require a method for the correct clinical diagnosis and ensure the proper treatment. These structures are considered one of the primary keys that detect and classify melanoma or nonmelanoma disease. Hence, we need an intelligent automated system to diagnose a patient's danger of malignant melanoma using dermoscopic images.

The need for a computerized skin cancer detection method is becoming one of the most challenging sciences. It is because of the detecting process, which is quite tricky. Also, the existing skin cancer images may contain noise such as hairs, shadows, specular reflections, and low contrast that reduce the image quality, which causes the accuracy of skin lesion segmentation algorithms to be less than clear images [4]. Active contour is a well-known method that can be used for segmentation. The active contour's primary purpose is to evolve a segmentation curve [5]. For example, to segment an object within the image, the curve of active contour starts from the initial point until it meets the object's boundary. Hence, starting with the angle around the object is the foremost important point in this method. It is the main drawback of the active contour method. This initialization problem also leads to low performance for concave boundaries and low performance when the contour is started far from the minimum. Hence, one of the main disadvantages of localizing active contour is the sensitive initial boundary to segment the object in the image. Some existing techniques proposed by hand allocate the initial point of seed to start active contour. Several methods carry out restarting if the first start does not return the object's accurate boundary of the object, so this is computationally expensive. The re-initialization process will maintain single object segmentation, while the initialization method is multiple object segmentation. However, the initialization method provides faster computational time than re-initialization. Therefore, we have proposed a method for initializing the seed point automatically, and it becomes more efficient and faster computational time than re-initialized. Sometimes, it also returns more than one object inside each other, which is another drawback.

This research paper will review existing techniques and propose a new skin cancer segmentation method.

### 1.1. Skin Anatomy Overview

**Figure 1:**
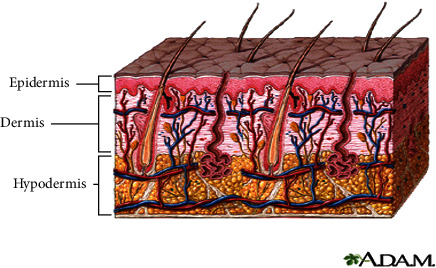
Skin layers.

The skin and its certain specialized derivatives called appendages constitute the integumentary system. The appendages include sweat glands, hairs, sebaceous glands, and nails. The skin (also called integument or cutis) forms the external covering of the body. It consists of two layers of completely different types of tissues attached over their entire extent. The superficial layer known as the *epidermis* consists of stratified squamous epithelium, while the deeper layer, called the dermis, is composed of dense connective tissue. Together, these two layers form a sheet that varies from 0.5 mm to 4.0 mm in thickness in different body parts [6]. The skin is generally classified into thick and thin types. The thick type covers palms and soles, while the thin skin is found on the remainder of the body. It is important to note that these terms, thick and thin, take into account the thickness of the *epidermis* only and do not refer to the thickness of the skin as a whole.  Figure 1 shows the basics of skin layers.

### 1.2. Epidermis

**Figure 2:**
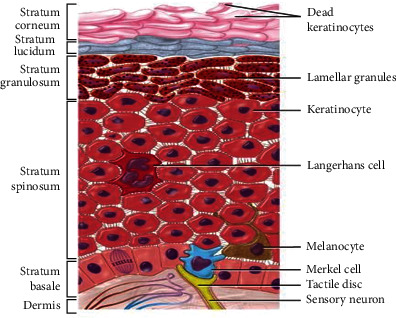
Epidermis layers and structures.

The *epidermis* is a continuously self-replacing laminated squamous keratinized epithelium. It contains four types of cells: melanocyte cells, keratinocyte cells, Langerhans cells, and Merkel cells.  Figure 2 shows the *epidermis* layers and structures.

### 1.3. Melanoma Growth Phase

The path of melanoma usually pursues two growth phases [7], namely, the vertical growth phase (VGP) and the radial growth phase (RGP). RGP generally leads to vertical growth phase. During the radial growth phase, cancerous cells spread outward in a radial pattern through the *epidermis* [8, 9]. At this moment, melanin and melanocytes are still constrained to the *epidermis* (that is to say, the skin cancer has not metastasized), so it is considered “in situ.” In order to achieve a favorable prognosis, this malignant cancer should be detected earlier when it is in its radial growth phase, earlier than cancer goes into a metastatic vertical growth phase. In VGP, melanocytes enter the dermis deeply and may invade the surrounding body tissues through metastatic events. This invasion of distant tissues is precarious since cancer can go wide in the body in several different components of the body [10, 11]. Lesions within the vertical growth phase frequently emerge as a nodule or bump on the surface. It is a hazardous phase, so lesions within this phase need to be immediately addressed.

## 2. Related Work

Regardless of current research to design decision support systems, there is much room to facilitate the scientists to explore and suggest efficient and effective decision support systems for acknowledgment in the field of medicine [12–14]. The method for the detection in the early stages is mainly concerned with the clinical characteristics utilized for image processing. These systematic characteristics provide valuable information to medical specialists, which assists them in analyzing melanoma patients. Computer-aided design (CAD) systems help physicians and practitioners to perform their responsibilities efficiently [15–17]. CAD systems are designed using visual features of images, but in selecting suitable features, major problems arise during the feature extraction stage. Therefore, this problem will be solved in our proposed research work to segment and classify the visual characteristics effectively.

Many techniques have been presented to segment skin lesion images automatically, but existing algorithms are only proposed for illumination. Segmentation procedures can be helpful to only those dermoscopy images, where there is a lot of difference between specific kinds of cancer and the surrounding skin area. Nawaz et al. [18] gathered a summary of new standard segmentation algorithms for dermoscopy images. The algorithms are compared with simple thresholding and region merging using the active contour method [19]. Many systems use only those characteristics obtained from pixel color [20]. The luminance channel from the CIE 1976 *L* ∗ *a* ∗ *b*^*∗*^ (CIELAB) or CIE 1976 *L* ∗ *u* ∗ *v*^*∗*^ (CIELUV) color spaces, the blue channel from the RGB color space, and an orthogonal transform are applied to these color channels. But, it is still a problem to segment the lesion parts correctly with undistinguishable boundaries when we rely only on color features. Such a method is not being operated on images of skin cancer.

In addition, some segmentation texture-based algorithms have been applied to dermoscopic images. Öztürk and Özkaya [21] have used some basic numerical methods, such as the gray-level co-occurrence matrix (GLCM), to recognize the texture or pattern of images of the skin body. This analysis can be helpful for the segmentation of the lesion parts and the classification of dermoscopic images. A textural-based algorithm for segmentation lesions using GLCM features was presented in [22]. Similarly, first-order region statistics and Sonali random field models were introduced. Sonali and Kamat [23] proposed a new method by using the natural skin texture sample on the four edges of the dermoscopic image. The presence of artifacts like shining areas and shadows caused by light makes the segmentation of skin lesion images more complicated. Hence, Manjubharathi and Saraswathi [24] explored more different algorithms, including thresholding, split-and-merge, and active contours, to change them to be specific for lesion images. The thresholding algorithm has to be changed to account for shining areas. Jeniva and Santhi [25] provided four different algorithms that modify the lighting variant of images before thresholding is applied to segmentation methods. Thresholding is used on multiple colors, a group of similar colors, or one-color channels derived by processing steps and primary component analysis [26]. Abbadi and Miry [27] proposed segmentation techniques, including the preprocessing stage, which assist in performing segments. Lu et al. [28] joined thresholding approach of segmentation for boundary lesion image with the form of fuzzy C-means to last segmentation process. Another algorithm was proposed based on the analysis of the NC ratio in segmentation by Baral, Gonnade, and Verma [29].

The experimental result shows high efficiency and robust segmentation process for a tumor cell. Khouloud et al. [30] applied the idea of texture distribution based on the learned model of natural human skin and cancer textures. Their results represented the difference between the texture distribution metric and captured the pair of texture distribution. The image is divided into many smaller parts based on similarity to achieve higher segmentation. Marchetti et al. [31] proposed the segmentation method to identify skin lesion cells in the superficial. The nuclei regions located on the superficial layer of skin are segmented with a *k* value equal to 3 using the K-means clustering techniques based on some color and space information [32]. Then, local region recursive technique segmentation is used to filter the interested nuclei regions to identify the area of nuclei which uses the intensity and size of nuclei as a parameter [33]. Finally, in the last stage, the local double ellipse descriptor is applied to discriminate melanocyte cells from keratinocyte cells.

The performance of the technique produced good results where the foreground and background both have the same form. Zhang et al. [34] suggested another Wiener filter technique to remove artifacts such as skin hair from the image. Then, they applied to thresholding to segment the skin lesion parts from the dermoscopic image. Experts in the medical domain compare the results of this segmentation method and measure the distance between these two results by using 96.32 percent accuracy of TDR and HM [33]. Gonzalez-Diaz [35] suggested segmenting melanocytes from the histopathological picture of the body. First, the local region's recursive algorithm and mean shift are used to remove nuclei bits [36].

Furthermore, a local double ellipse descriptor incorporated melanocyte characteristics that provide melanocyte recognition parameters by using 30 histopathological skin objects with different features, such as a slice. This methodology showed a sensitivity rate of approximately 80% and a successful prediction rate of roughly 70% [37]. Li et al. [38] presented a segmentation method based on the neuro-fuzzy model. Segmentation is achieved by operating as parameters with some functions. This approach provides good accuracy, robust quality, and accuracy [39].

## 3. Proposed Method

The proposed method joins many phases and strategies to improve segmentation outcomes' accuracy and robustness. The segmentation processes will be performed to detect and segment the lesion parts from the skin automatically. To segment the image, the following are the details:

(1) Converting color images into gray images
(2) Creates Gaussian filters using
  (1) $$\begin{array}{c} h_{g}\left(n_{1},n_{2}\right)=\;e^{\frac{-\left(n_{1}^{2}+\;n_{2}^{2}\right)}{2\sigma^{2}}}, \end{array}$$
  (2) $$\begin{array}{c} h\left(n_{1},n_{2}\right)=\;\frac{h_{g}\left(n_{1},n_{2}\right)}{\sum_{n_{1}}\sum_{n_{2}}h_{g}}, \end{array}$$
equations (1) and (2), *n*_1_ and *n*_2_ are the pixel values of the image, *σ* is the standard deviation, and *h* is the value of the Gaussian filter.
(3) Calculate threshold by using maximum entropy calculated by the following mathematical equations:
equation (3), *h* (*i*) is a normalized histogram value, which takes integer values from 0 to 255. Hence, assume that *h* (*i*) is a normalized value, Eq. (4) shows the entropy of white pixels, Eq. (5) shows entropy of black pixels, and Eq. (6) maximizes white and black pixels' optimal threshold which can be selected:
  (3) $$\begin{array}{c} \sum_{i=0}^{i_{max}}h\left(i\right)=1, \end{array}$$
  (4) $$\begin{array}{c} H_{B}\left(t\right)=-\overset{i}{\underset{i=0}{\sum }}\frac{h\left(i\right)}{\sum_{j=0}^{t}h\left(j\right)}\;log\frac{h\left(i\right)}{\sum_{j-0}^{t}h\left(j\right)}, \end{array}$$
  (5) $$\begin{array}{c} H_{B}\left(t\right)=-\overset{i}{\underset{i=i+1}{\sum }}\frac{h\left(i\right)}{\sum_{j=i+1}^{t}h\left(j\right)}log\frac{h\left(i\right)}{\sum_{j=i+1}^{t}h\left(j\right)}, \end{array}$$
  (6) $$\begin{array}{c} T=Arg_{i=0\ldots \;i_{max}}Max\;H_{b}\left(t\right)+H_{w}\left(t\right). \end{array}$$
(4) Apply median filter to reduce noise and preserve edges.
(5) To remove tiny parts from images after thresholding, all connected components or objects with fewer pixels from the binary image need to prune. Thus, the operation has been performed using eight connectivity components known as an area opening.
(6) Morphology operations opening and closing have been applied.
(7) Adaptive contour method is based upon the optimal mask to segment the skin lesion.
(8) Refinement of segmentation using morphology operations has been performed.
(9) Remove small holes outside the body.
(10) Select one big area that shows a lesion.

**Figure 3:**
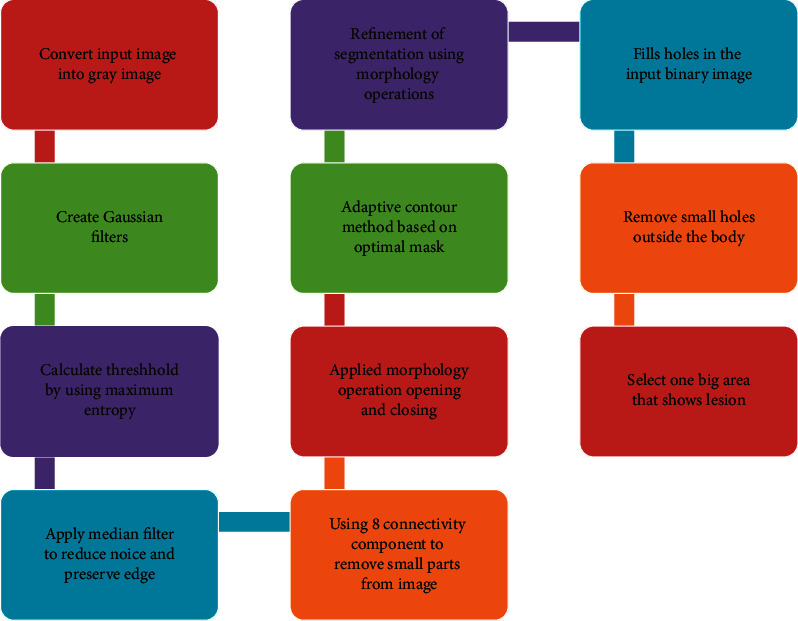
Proposed method.

This paper uses the adaptive contour (snack) segmentation method. The initialization mask is the most important for active contouring methods to feed the seed point required in that algorithm. One way is to select this mask manually for seed point, but it is not easy to apply to all types of images. Thus, it is required to propose a method shown in Figure 3 for the selection of masks automatically using the active contouring method. We have proposed a technique based on the Laplacian Gaussian filter-based maximum entropy for mask selection. Details of these steps are explained below.

According to many previous studies and research, there are many methods to perform the threshold process. After testing those methods in the actual experiments, they may provide accurate results in some cases, but they failed in many of them due to the different nature of the images. The main issue the existing algorithms faced was the low quality of the picture. Noise is sometimes present in the form of hair or shadow, which affects their results. Those methods must either choose the initial threshold value, which is a big problem, or use a calculated average weight as an initial threshold. Using average value may fail in low-quality/noisy images since the average is not robust to noise. So, we used Laplacian Gaussian filter-based maximum entropy technique that gives a dynamic and the optimal threshold.

This stage aims to provide a dynamic, optimal, and adaptive threshold. That threshold will be used in the advanced stage toper from the segmentation of the image.

Morphological operations have been used to detect the best seed point and for a clear map representing the lesion in the image. It uses the functions of morphology like opening and closing operations and the structure/synthesized operations to produce a better picture. Many factors affect the accuracy of that algorithm's output, like the characteristic of structuring elements and synthesized modes of the functions. In more detail, it can consider the synthesized model of operators reflects the relationship between the processing image and the original one and choosing the synthesized mode will affect the accuracy and the outcome. Hence, the secrets of morphological operations will be simplified for the morphological filter structure design and the selection of structuring elements. To detect the border on the medical images, we have to choose the appropriate structure element by the texture features of the picture. Factors like the shape, direction of structuring elements, and size should be considered.

In most cases, the structuring element is selected to be 3 ∗ 3 square. Dilation, erosion, opening, and closing are primary operations and functions of binary morphology. We chose the disk-structuring element using the empirical method, the size of the structuring element used for opening is nine, and for closing, it is 25.

Kass introduced the central concept and the idea of the active contours “Snakes: Active Contour Model.” The definition of the snake is a dynamic curve based on the parameter, which attempted to move into a minimum energy position. The snack energy can be calculated using the energy function shown in (7) and (8), which Kass introduced.

(7) $$\begin{array}{c} E_{snake}^{*}=\int_{0}^{1}E_{snake}\left(v\left(s\right)\right)ds, \end{array}$$

(8) $$\begin{array}{c} E_{\text{snake}}^{*}=\int_{0}^{1}E_{snake} \end{array}$$

The energy of the snake segmentation algorithm consists of three essential terms. “*E*_int_” denotes the snake's internal power, “*E*_img_” represents the forces of the image, and “*E*_con_” rises to external constraint forces. The external snake forces (*E*_ext_) can be calculated by summing *E*_img_ and Econ values. The parametric curve “C” is the classical snake used in this research. It is attracted to the area with intense gradients where the typical gradient weight of any point is high. The principle of snake segmentation is to locate the initial contour in the image, which is distorted under various energies. There is a need for two kinds of energy, i.e., internal and external.

Internal energy: It depends on the parametric curve's first and second derivatives representing the snake. This energy depends on the main properties of the curve and the sum of bending and elastic energy. (9) shows the internal energy equation.

(9) $$\begin{array}{c} E_{int}=E_{elastic}+E_{bending}=\int_{0}^{1}\frac{1}{2}\left(\alpha |\left|v_{s}\right|{|}^{2}+\beta {\left|v_{ss}\right|}^{2}\right)d. \end{array}$$

The external energy relates to image characteristics, such as the availability of noise or edges in the image. It guarantees that the snake is on the edge when maximizing the standard gradient's amount over the curve and thus minimizing its opposite. External energy (*E*_ext_) of the contour comes from the image and takes on its smaller parts of the function of, for example, boundaries. (10) shows the external energy equation.

(10) $$\begin{array}{c} E_{ext}=\int_{0}^{1}E_{image}\left(v\left(s\right)\right)ds\;. \end{array}$$

First, the initial contour is in the center of the circle in the image. Then, the iteration of the algorithm will move; this will cause the total energy to be reduced. A function *E* will be computed for every neighborhood point (*n*) of active contour energy point (*p*). The point p0 attempts to minimize the energy E0 and then exchange contour point P if *E* > *E*0. Otherwise, it will keep it the same. This operation will be repeated until convergence when the contour gained at iteration *t* equals that gained at iteration *t* + 1.

After applying the adaptive contour method, sometimes it produces more than one segment inside each other. Thus, it is essential to fill the big-segmented part. We have used eight connected neighborhoods to fill the larger segment.

## 4. Results and Discussion

We have evaluated the performance of our proposed technique using a dataset of 69 dermoscopic images. These images have been gathered from DermIS database. These images belong to the two primary melanoma and nonmelanoma classes. The dataset we have used contains 43 images of melanoma and 26 images of nonmelanoma. The result of the proposed segmentation is shown in Table 1. The original image column shows the input image from DermIS dataset. The second column, segmented images, shows theoutput after applying the segmentation method. The boundary detection column shows the detection of the boundary of the lesion in that image.

Table 2 shows the original and segmented images after applying the proposed segmentation technique. Some morphology operations have been applied to correct the images and select the mask for the adaptive contour method. We have used the adaptive contour method for segmentation. The adaptive contour method requires one mask as a seed point to start for segmentation, and then it grows with every iteration until the stopping condition meets. First, Gaussian filter-based maximum entropy has been used to calculate the adaptive and optimal threshold. Based upon this threshold, the image has been segmented, and then morphology operations opening and closing have been applied to select a specific mask for the adaptive contour method.

Thus, this mask gives a seed point to start the adaptive method. Column 1 shows the original images. Column 2 shows the segmented binary image after applying the proposed method for segmentation. In the third column, the segmented part is highlighted in green color in the original image. These images clearly show that the proposed method works for all images perfectly. The primary reason behind this good segmentation is finding the optimal mask for active contour. Segmentation results are outstanding and effective if the extracted mask is good.

In the previous phase, the segmentation was performed on dermoscopic images to extract the portion of the lesion. The proposed technique is described in the last section in detail. In this section, only the experimental results are shown.

The segmentation performance is measured and validated by the area base correlation criteria and Dice similarity coefficient frequently used in the literature. These criteria are given below.

### 4.1. Jaccard Similarity Index for Region Coincidence

The performance of the segmentation method is measured by the Jaccard similarity index based on region coincidence criteria [35, 36]. The following formula calculates the accuracy of the segmentation as in

(11) $$\begin{array}{c} P\left(R,A\right)=\frac{\left|A\cap R\right|}{\left|A\cup R\right|},0\leq P\left(R,A\right)\leq 1\text{,} \end{array}$$

where A is the ground truth structure, the *R* segment is the detected foreground structure, and A represents the segment area computing operation. Factor |*A* ∪ *R*| shows how much ground truth structure is detected. The factor |*A* ∪ *R*| shows the normalization, which normalizes the accuracy measure. The value 1 indicates the ideal similarity or matching between the computed area by the system and ground truth.

### 4.2. Dice Similarity Coefficient

Dice coefficient (Dice, 1945) measures the spatial overlap of two sets. In the proposed system, this method is used to validate the performance of the segmentation method. In our case, the one set is the segmentation by the proposed method, and the other group is the ground truth segmentation by the expert. The Dice similarity coefficient is defined as in

(12) $$\begin{array}{c} DS\;C_{\left(A,R\right)}=\frac{2\left|A\cap R\right|}{\left|A\right|+\left|R\right|}. \end{array}$$

A indicates the pixels of segmentation extracted by the proposed system, and *R* is the area by the expert as a ground truth. The value of DSC ranges from 0 to 1. The ideal weight of DSC is 1; i.e., the performance of the segmentation method agrees if DSC values are closer to 1.

Tables 3 and 4 show our proposed methodology result performance. The performance of the segmentation process is frequently measured and validated by the region base coincidence criteria and Dice similarity coefficient. The value 1 indicates the ideal similarity or matching between the computed area by the system and ground truth. This approach provides good accuracy and is robust. The segmentation part can be used for feature extraction to classify skin lesions..

Comparison has been made with different existing algorithms used for dermoscopic images.  Table 5 shows that the proposed method performs well using all performance measures. Dice coefficient (Dice, 1945) measures the spatial overlap of two sets and shows 0.969. Similarly, Jaccard similarity index for region coincidence also shows the best result 0.969. Both parameters show the best result. Our method has higher segmentation performance and capability compared with previous method.

## 5. Conclusion and Future Work

This research presents an automated and novel set of approaching techniques for the segmentation of dermoscopic images using automated and intelligent methods. Proper segmentation of skin cancer melanoma and subsequent diagnoses using automatic computer-aided design (CAD) systems is not only a trending research phenomenon but also a necessity of modern times. This objective was met by developing techniques and combining them within one framework, which not only promised efficiency. But, it also achieved better results than the existing solutions to this problem.

This research proposes a new skin lesion segmentation method by combining Gaussian filter-based maximum entropy-based threshold for mask selection, morphology operations, and adaptive contour method. We use Jaccard similarity index for region coincidence and Dice similarity coefficient to evaluate this method. Our method shows better results when comparing it with the image segmented by the expert dermatologist. Thus, the major contribution is to propose a mask selection for active contour and morphology operations as a preprocessing and postprocessing of active contour.

In the future, if we will combine preprocessing and postprocessing stages which extract the features and classify the input skin cancer images into melanoma and nonmelanoma using a machine learning classifier, our modal performance will be better on all medical application.

## Figures and Tables

**Table 1:** Summary of existing segmentation methods.

Reference	Approach	Disadvantages
Stoecker et al. [15]	Gray-level co-occurrence matrix for texture feature	The presence of artifacts like shining areas and shadows caused by light makes the process of segmentation of skin lesion images more complicated.

Stoecker and Scharcanski [22]	Four different algorithms	To identify the region of nuclei which used the intensity and size of nuclei as a parameter

Sonali and Kamat [23]	Combined thresholding with fuzzy C-means	It may not perform well over images with huge variations in skin colors

Manju Bharathi and Sarswati [24]	NC ratio analysis for automatic segmentation of cells	Performance degrades over lesions of varying sizes and shapes

Jeniva and Santhi [25]	Learning model of natural skin texture and cancer textures	A lot of difference between specific kinds of cancer and the surrounding area of skin

Kumar et al. [26]	Local region recursive segmentation, K-means clustering, and local double ellipse descriptor	It may not perform well over images with huge variations in skin colors

Abbadi and Miry [27]	Thresholding and Wiener filter	Low lesion-to-skin gradient, depigmentation, multiple tumor regions

Lu et al. [28]	Mean shift, local region recursive segmentation, and local double ellipse descriptor	This method is computationally complex

Baral, Gonnade, and Verma [29]	Neuro-fuzzy model and some other features	Complex thresholding approaches

**Table 2:** Original images, segmented, and boundary detected.

Original image	Segmented images	Boundary detection
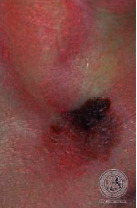	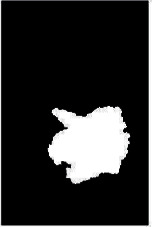	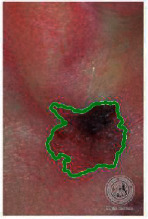
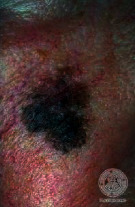	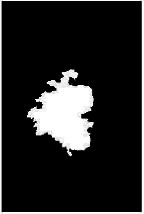	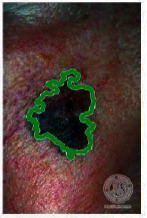
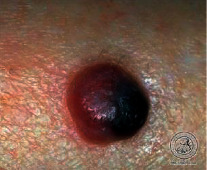	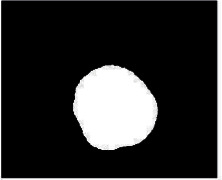	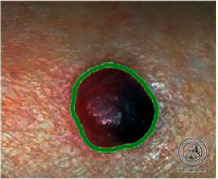
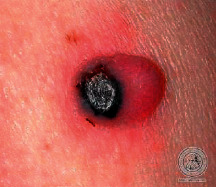	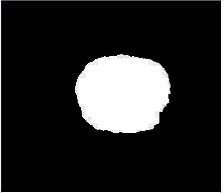	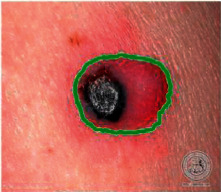

**Table 3:** Accuracy measure of segmentation with ground truth from melanoma images.

Dataset	Data size (pixels)	|**A**∩**R**| (Pixels)	|**A** ∪ **R**| (Pixels)	P (out of 1)	DSC (out of 1)
Image# 6	226 × 276	835	845	0.967	0.963
Image# 7	226 × 210	1794	1707	0.955	0.954
Image# 8	226 × 276	1543	1569	0.934	0.931
Image# 9	226 × 210	1181	1189	0.924	0.952
Image# 10	224 × 275	1303	1321	0.944	0.931
Image# 12	224 × 275	2154	2171	0.931	0.923
Image# 14	226 × 275	1769	1784	0.947	0.951
Image# 15	226 × 276	2711	2743	0.941	0.942
Image# 16	224 × 276	3049	3064	0.934	0.932
Image# 27	224 × 210	1628	1643	0.942	0.943

**Table 4:** The accuracy measure of segmentation with ground truth from nonmelanoma images.

Dataset	Data size (pixels)	|**A**∩**R**| (Pixels)	|**A** ∪ **R**| (Pixels)	P (out of 1)	DSC (out of 1)
Img44	226 × 276	10023	10103	0.945	0.944
Img45	226 × 210	14324	14379	0.963	0.969
Img46	226 × 276	3145	3304	0.952	0.958
Img47	226 × 210	3823	4011	0.943	0.951
Img48	224 × 275	359	367	0.927	0.923
Img49	224 × 275	2768	2731	0.946	0.941
Img50	226 × 275	466	456	0.933	0.939
Img51	226 × 276	4167	4107	0.925	0.927
Img52	224 × 276	2572	2519	0.921	0.928
Img53	224 × 210	933	956	0.938	0.932

**Table 5:** Comparing results with existing methods.

Method	Nonmelanoma images		Melanoma images	
	DSC (out of 1)	P (out of 1)	DSC (out of 1)	P (out of 1)
Long et al. [40]	90.46	82.59	89.03	80.22
Badrinarayanan et al. [41]	91.32	84.03	9 84.55	73.23
Ronneberger et al. [42]	89.88	81.63	82.04	69.55
Al-Masni et al. [43]	91.38	84.13	92.92	86.77
Proposed method	0.969	0.963	0.963	0.967

## Data Availability

The data used in this research will be available upon request from the corresponding author.
